# Laparoscopic-assisted Soave procedure for Hirschsprung disease: 10-year experience with 106 cases

**DOI:** 10.1186/s12893-022-01528-9

**Published:** 2022-02-26

**Authors:** Yun-jin Wang, Yuan-bin He, Liu Chen, Yu Lin, Ming-kun Liu, Chao-ming Zhou

**Affiliations:** Department of Pediatric Surgery, Fujian Maternity and Child Health Hospital, Affiliated Hospital of Fujian Medical University, Fuzhou, 350001 People’s Republic of China

**Keywords:** Long-segment, Hirschsprung disease, Soave procedure, Laparoscope

## Abstract

**Background:**

The purpose of this study was to summarize the clinical experience and 10 year follow-up results of laparoscopic assisted Soave procedure for the treatment of long-segment Hirschsprung disease (HD).

**Methods:**

From January 2010 to February 2020, 106 children with long-segment HD participated in this study. The laparoscopic-assisted Soave procedure was performed for the treatment of long-segment HD. The follow-up time was two weeks, one month, and three months after the operation, and then every six months to one year.

**Results:**

The operation was successful for all 106 children. All patients were discharged 5–7 days after the operation. The median time in surgery was 150 (100–190) minutes, and the median volume of bleeding was 6 (3–10) ml. The short-term postoperative daily defecation frequency was 4–11 times, 3–7 times within 6 months, and 2–3 times after 6–12 months. Postoperative complications included anastomotic leakage in two cases, perianal dermatitis in 13 cases, anastomotic stenosis in four cases, adhesive bowel obstruction in two cases, enterocolitis in 16 cases, soiling in 11 cases, and constipation recurrence in three cases.

**Conclusions:**

The laparoscopic-assisted Soave procedure is a safe and effective surgical method for treating long-segment HD, and it causes little trauma or bleeding and has a fast postoperative recovery. Yet some complications may occur. Preoperative diagnosis, intraoperative and postoperative standardized processing can reduce the postoperative complications.

## Background

Hirschsprung disease (HD), also known as intestinal aganglionosis [1], is the most common congenital enteric neuropathy despite an incidence of only 1/5000 births [2]. It is caused by a defect in the cranial-caudal migration of vagal neural-crest cells along the intestine during early embryonic development [3], resulting in the absence of ganglion cells in the muscular layer and submucosa of the distal intestine, persistent spasms of the distal intestine, compensatory hypertrophy and expansion of the proximal intestine, and symptoms including constipation, abdominal distention, and intestinal obstruction [4, 5]. It seriously affects the quality of life and the growth of children, and resection of the affected bowel by surgical methods is the most used treatment for HD [6]. However, the traditional open radical operation causes trauma to the child, and the recovery is slow. Due to the serious trauma and long postoperative recovery time, children are susceptible to many other complications [7]. With the improvement of laparoscopic instruments and surgical technology, the use of minimally invasive treatment is a developing trend for treatment of HD. Laparoscopic-assisted surgery is suitable for long-segment, total-colon HD and its allied disorders. It has the advantages of less trauma, a simple operation with greater safety and effectiveness, and fewer complications [8–10]. In recent years, the laparoscopic-assisted Soave procedure has been widely and successfully used in several nations. In this paper, we review the clinical data of 106 children with long-segment HD treated with the laparoscopic-assisted Soave procedure in our hospital.

## Methods

This study was approved by the ethics committee of Fujian Maternity and Child Health Hospital, Affiliated Hospital of Fujian Medical University, and strictly adhered to the tenets of the Declaration of Helsinki. All patients’ guardians signed an informed consent form before the operation.

### Patients

The clinical data of 106 patients with long-segment HD from our hospital from January 2010 to February 2020 were analyzed retrospectively. This included preoperative, intraoperative, postoperative data, and follow-up data. The aganglionic level above the sigmoid colon constitutes long‐segment HD. Inclusion criteria: patients presented with long-segment HD. Exclusion criteria: (1) total colon aganglionosis, age of operation ≥ 14 years, or Down's syndrome; (2) patient with a poor overall physical condition, such as having severe hepatic or renal insufficiency; (3) refused to sign the consent form for surgery or refused to comply with the follow-up schedule.

All children had a history of delayed excretion of meconium and long-term constipation. Preoperative diagnosis was made based on barium enema, positive and lateral X-ray films of 24 h residual barium, anorectal manometry and suction rectal biopsy. The diagnosis was further confirmed by intraoperative frozen biopsy and reconfirmed postoperatively by conventional histopathology on the resected specimen. All patients, 66 males and 40 females, were positively diagnosed with long-segment HD. The patients were from 1 month to 7 years old (Table 1). All patients received colonic irrigation for 7–14 days after admission to excrete residual feces and reduce intestinal dilatation, and some patients were given colonic irrigation for 3-weeks to improve their nutritional status and treat related complications. Oral antibiotic treatment was performed for bowel preparation for three days and intravenous antibiotics 30 min before the operation.

**Table 1:** Clinical data of the patients in this study

Item	
Number of patients	106
Boys/Girls	66/40
Age, median (range)	2 years (1 month-7 years)
Weight,median (range)	13.5 (4.5–24)kg
Operation time, median (range)	150 (100–190) min
Volume of bleeding, median (range)	6 (3–10) ml
Time of discharge postoperative, median (range)	5.5 (5–7)days
Duration of follow-up, median (range)	5.3 years (3 month-10.2 years)

### Technique

**Fig. 1:**
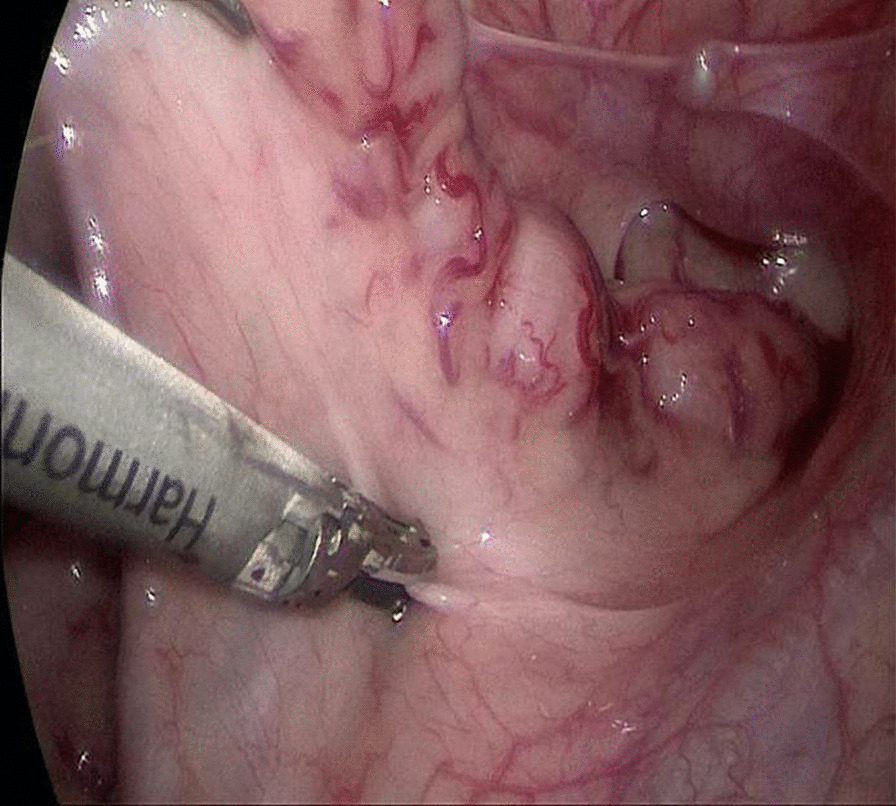
The sarcoplasmic tissue of the upper rectum wall was cut under laparoscopy

An approximately 5 mm layer-by-layer incision was made in the umbilical skin, and a 5 mm trocar was placed directly into the abdomen to establish a pneumoperitoneum (12 mmHg). Under the guidance of laparoscopy, two trocars for the forceps and the ultrasonic scalpel were placed at the lateral right and the left rectus abdominis muscle at the umbilical level. We then determined whether the upper rectum and sigmoid colon were spasmodic, the lower descending colon was dilated, the intestinal wall was thickened like leather, the colonic pouch had disappeared, and if peristalsis was poor. The sarcoplasmic tissue of the upper rectum wall was cut under laparoscopy (Fig. 1), and a 1 × 1 cm section was sent for frozen pathology during the operation. When the pathologist reported no ganglion cells found, we then diagnosed long-segment HD. The aganglionic segment was mobilized circumferentially distally to approximately 2 cm below the peritoneal reflection and proximal to the bowel with a size similar to that of the normal bowel. Seromuscular samples of about 1 × 1 cm of the proximal bowel with normal appearance were taken for frozen biopsies until ganglion cells were found. This procedure was continued for the proximal ganglionic bowel that could be pulled deep into the pelvis without tension. We then expelled the air from the abdominal cavity and the procedure was continued with the anus operation.

**Fig. 2:**
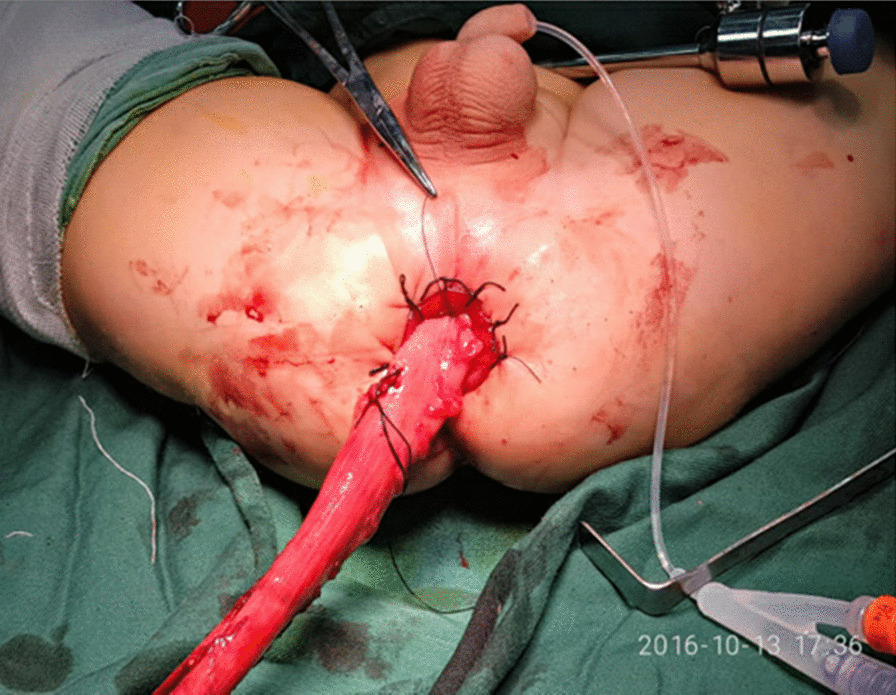
The affected bowel was pulled out of the anus without torsion or tension

**Fig. 3:**
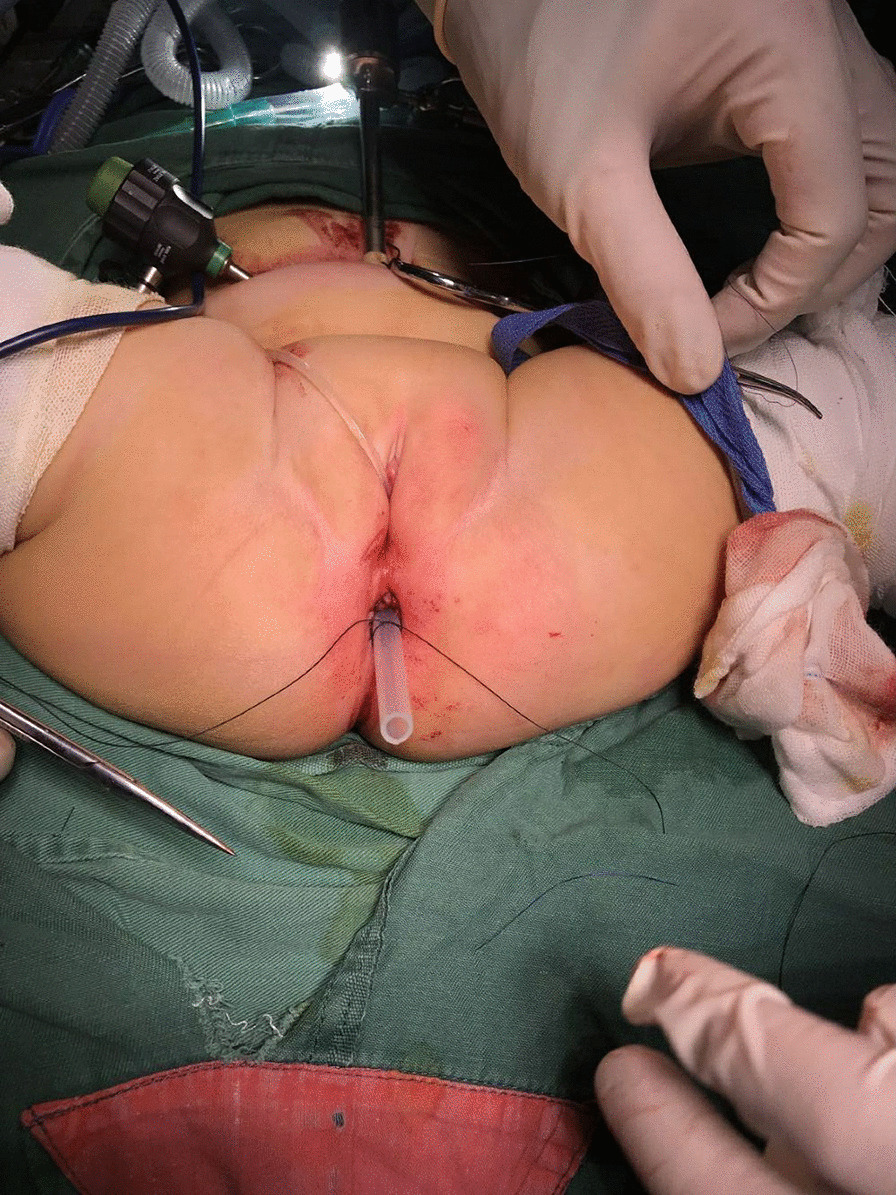
We inserted a 10 cm tube into the anus for drainage and kept it in for 3–7 days

About 0.5-1 cm above the dentate line of the rectum an oblique circumferential incision of the rectal mucosa was made, and the rectum muscular sheath of the posterior wall was removed to reach the level of the retroperitoneal fold. A circular incision was performed in the rectal muscular sheath to connect with the abdominal cavity. The affected bowel was pulled out of the anus without torsion or tension (Fig. 2). The aganglionic and dilated segments were resected. Coloanal anastomosis was performed with interrupted fine absorbable sutures. The pneumoperitoneum was established again to check that there was no active bleeding in the abdominal cavity and no torsion of the pulled colon. The peritoneal gas was released, and we removed the trocar before suturing the umbilical incision. Finally, we inserted a 10 cm tube into the anus for drainage and kept it in for 3–7 days (Fig. 3).

## Results

No child had a stoma before the laparoscopic-assisted Soave procedure for HD. This might be related to our adequate preoperative preparation. All 106 children with long-segment HD successfully underwent the laparoscopic-assisted Soave procedure. The aganglionic segment was located in the ascending colon in 23 patients, in the transverse colon in 25 patients, and in the sigmoid or descending colon in 58 patients. All patients were discharged 5–7 days after the operation. The median operation time was 150 (100–190) minutes (Table 1). Anal dilatation was performed 14 days postoperatively on all patients and lasted for 6–8 months. The frequency of daily defecation postoperatively was 4–11 times within 1 month. After 6–12 months the defecation frequency was 2–3 times per day.

After the operation, anastomotic leakage occurred in two patient and perianal dermatitis in 13 patients. Four patients suffered anastomotic stenosis, all of whom responded well to anal dilatation. Two patients developed postoperative adhesive bowel obstruction, which were mild and resolved using conservative treatment. There were 16 patients who had complications of Hirschsprung-associated enterocolitis (HAEC), but resolution occurred with anti-infection steps and colon irrigation. In 11 cases of soiling, 8/11 cases were cured, and 3/11 cases were improved after prolonged anal dilatation, regular sitting baths, and defecation training. Three patients who had recurrent constipation were successfully treated using glycerin for defecation or colon irrigation.

The follow-up period was three months to 10.2 years, and the median follow-up time was 5.3 years. The follow-up intervals were two weeks, one month, and three months after the operation, then every six to 12 months. The content of the follow-up included the symptoms and signs that were obtained from outpatient visits or detailed telephone interviews.

## Discussion

The main clinical manifestations of HD are dyssynergic defecation, gastrointestinal dysfunction, and malnutrition, which affect the healthy growth of children. Surgery is an effective method for the treatment of HD. The traditional open radical operation has a long operation time, serious trauma, many postoperative complications, and high mortality [11]. It leaves an obvious abdominal scar and there is a long exposure time of abdominal organs, which can easily cause more bleeding during the operation and adhesive intestinal obstructions after operation that can delay the start of eating and prolong the hospital stay [12]. With the evolution of medical technology laparoscopy has become an important diagnosis and treatment method for minimally invasive surgery [13, 14]. Excision of the aganglionic bowel can be performed using many procedures, such as the Duhamel, Swenson, Soave, and Rehbein procedures [15]. Laparoscopic Swenson procedure is a technically difficult operation with a high rate of injury to pelvic structures [16]. It is associated with significant injuries with considerable bleeding and is prone to damage the nerve, resulting in postoperative neurogenic bladder. Laparoscopic Duhamel is relatively complex and requires suture cutting equipment [17]. In recent years, the laparoscopic Soave has become popular in clinical practice due to its advantages of minimal invasion and good efficacy.

Compared with conventional open surgery, the laparoscopic Soave has the following advantages: First, the operation can reduce the damage to the deep pelvic tissue of the child by separating the rectum from the peritoneum and then pulling out the aganglionic part of the bowel. Second, it is easy for surgeons to observe the structure of the abdominal cavity and pelvic cavity from multiple directions and perspectives [18], and to deal with any hidden disease or condition in the abdominal cavity at the same time. It is easier to clearly judge the scope of the aganglionic segment, and observe whether there is torsion, monitor the blood supply to the intestinal tract and any abdominal bleeding, or other conditions after anastomosis of the ganglionic segment at the anus, to improve the precision of the operation. Third, it can reduce the risk of external anal sphincter injury, improve the newly-built intestinal fecal storage and defecation control function, reduce the incidence of soiling and constipation, and promote the postoperative recovery [19]. Fourth, it uses a small incision, is minimally invasive, and has a good cosmetic effect. This approach pulls the abnormally innervated bowel out of the anus for direct observation during resection, reducing the contamination to the abdominal cavity and avoiding intestinal exposure. Therefore, the bowel peristalsis function recovers quickly after laparoscopic surgery, reducing intestinal adhesions and intestinal obstruction. In this study, all patients began to eat a liquid diet 2–3 days after their operation and were discharged at 5–7 days.

Although the laparoscopic Soave has many advantages, there are some disadvantages to this operation. First, it requires a certain learning curve. In addition, it needs special laparoscopic instruments, and the cost of hospitalization is high. Due to the abdominal space for laparoscopic operation being small and the surgical instruments being small, some long-segment HD needs to be reversed during the operation. This technique also requires significant laparoscopic experience. It is the most difficult step and a key step of the whole operation. The ascending colon should be rotated 270° counterclockwise so that the ileocecal part is located in the pelvic cavity. To monitor the whole process of pulling out and rotating under the laparoscopic, the function of the abdomen and perineum should cooperate with each other to make the rotation angle between the abdominal and the anorectal intestine consistent. Applying this method, we found no torsion in any case and good results were obtained.

Whether a traditional abdominal approach or laparoscopic approach is performed, there are problems with postoperative constipation recurrence. The causes of constipation recurrence after laparoscopic surgery include mechanical obstruction, persistent or acquired aganglionosis, transitional segment residue, intestinal motility disorder, and so on [20]. Constipation recurrence still occurs in 8%-30% of children following a radical operation for HD [21]. In this study, there were three (2.8%) cases of constipation that occurred who were successfully treated using glycerin for defecation or colon irrigation. Our experience is that under the direct vision of laparoscope, we can check the tightness of the pulled-through intestine, observe the blood supply, avoid the occurrence of distal intestine ischemia, and remove the aganglionic bowel completely.

Anastomotic stenosis is also a common complication after operation of HD. Standardized anal dilation exercise can effectively prevent the occurrence of anastomotic stenosis. Subsequently, a dilation program was started 14 days postoperative, parents were taught to continue this program at home after discharge to avoid anastomotic stenosis and constipation recurrence. Generally, the anal dilatation was started with a No. 6 or 7 (6 mm or 7 mm, 1 mm is a unit in size) anal dilators. The size of the anal dilators was increased every two weeks for at least 6 to 8 months [22]. In this study, there were four (3.8%) cases of anastomotic stenosis that occurred who did not continue this program due to poor parental compliance that were cured after anal dilatation treatment.

HAEC is the most frequent serious complication following a radical operation for HD [23]. It has been reported that the preoperative incidence of HAEC is 6%-60%, and the postoperative incidence is 25–37% [24]. Umeda et al. reported that food allergy, genetic factors, imbalance of intestinal microecology, abnormal secretion of mucin or immunoglobulin and abnormal function of intestinal nervous system can lead to enterocolitis^25^. There were 16 (15.1%) cases of HAEC in our group, lower than the incidence reported in the literature. We consider that in addition to colon obstruction and other factors, it is mainly related to the intestinal immune mechanism of children. Abnormal intestinal immune function is also one of the causes of enterocolitis.

This retrospective study has several limitations. First, this was a single-center study with a limited number of patients. Second, this study was a retrospective review without a comparison group nor statistical analysis. Large, randomized studies with long-term follow-up are necessary to assess the effectiveness and complications of this technique in further studies.

## Conclusion

In conclusion, laparoscopic-assisted Soave procedure is a safe and effective method for treating long-segment Hirschsprung's disease. This method has the advantages of little trauma or bleeding, and a fast postoperative recovery. Yet some complications may occur. Preoperative diagnosis, intraoperative and postoperative standardized processing can reduce the postoperative complications.

## Data Availability

The datasets generated during and analysed during the current study are not publicly available due to patient privacy but are available from the corresponding author on reasonable request.

## Abbreviations

HD: Hirschsprung disease
HAEC: Hirschsprung-associated enterocolitis
